# Cranberry (*Vaccinium macrocarpon*) Extract Impairs Nairovirus Infection by Inhibiting the Attachment to Target Cells

**DOI:** 10.3390/pathogens10081025

**Published:** 2021-08-13

**Authors:** Mattia Mirandola, Maria Vittoria Salvati, Carola Rodigari, K. Sofia Appelberg, Ali Mirazimi, Massimo E. Maffei, Giorgio Gribaudo, Cristiano Salata

**Affiliations:** 1 Department of Molecular Medicine, University of Padova, 35121 Padova, Italy; mattia.mirandola@gmail.com (M.M.); mariavittoria.salvati@phd.unipd.it (M.V.S.); carola.rodigari@studenti.unipd.it (C.R.); 2 Department of Microbiology, Public Health Agency of Sweden, 17182 Solna, Sweden; sofia.appelberg@folkhalsomyndigheten.se (K.S.A.); ali.mirazimi@ki.se (A.M.); 3 Department of Laboratory Medicine, Karolinska Institutet, 17177 Stockholm, Sweden; 4 National Veterinary Institute, 75189 Uppsala, Sweden; 5 Department of Life Sciences and Systems Biology, University of Turin, 10123 Turin, Italy; massimo.maffei@unito.it (M.E.M.); giorgio.gribaudo@unito.it (G.G.)

**Keywords:** Hazara virus, Crimean-Congo hemorrhagic fever virus, *Vaccinum macrocarpon* extract, A-type proanthocyanidins, virucidal and antiviral activities

## Abstract

Hazara virus (HAZV) belongs to the *Nairoviridae* family and is included in the same serogroup of the Crimean-Congo hemorrhagic fever virus (CCHFV). CCHFV is the most widespread tick-borne arbovirus. It is responsible for a serious hemorrhagic disease, for which specific and effective treatment and preventive systems are missing. Bioactive compounds derived from several natural products may provide a natural source of broad-spectrum antiviral agents, characterized by good tolerability and minimal side effects. Previous in vitro studies have shown that a cranberry (*Vaccinium macrocarpon* Ait.) extract containing a high content of A-type proanthocyanidins (PAC-A) inhibits the replication of herpes simplex and influenza viruses by hampering their attachment to target cells. Given the broad-spectrum antimicrobial activity of polyphenols and the urgency to develop therapies for the treatment of CCHF, we investigated the antiviral activity of cranberry extract against HAZV, a surrogate nairovirus model of CCHFV that can be handled in Level 2 Biosafety Laboratories (BSL-2). The results indicate that the cranberry extract exerts an antiviral activity against HAZV by targeting early stages of the viral replication cycle, including the initial adsorption to target cells. Although the details of the molecular mechanism of action remain to be clarified, the cranberry extract exerts a virucidal effect through a direct interaction with HAZV particles that leads to the subsequent impairment of virus attachment to cell-surface receptors. Finally, the antiviral activity of the cranberry extract was also confirmed for CCHFV. As a whole, the evidence obtained suggests that cranberry extract is a valuable candidate to be considered for the development of therapeutic strategies for CCHFV infections.

## 1. Introduction

The Hazara virus (HAZV) is a member of the *Orthonairovirus* genus of the *Nairoviridae* family [1]. The *Nairoviridae* family comprises 45 tick-borne enveloped arboviruses, with three circular negative-stranded RNA genome segments, named as small (S), medium (M) and large (L), based on their nucleotide length. These vRNAs encode the nucleoprotein (NP), the glycoproteins precursor (GP), and the RNA-dependent RNA polymerase (RdRP), respectively. Each vRNA is encapsidated by NP units and interacts with one RdRP molecule, forming a viral ribonucleoprotein (vRNP) complex [1].

After the envelope glycoprotein-mediated adhesion to unknown cell-surface receptor(s), nairovirus particles are internalized through the endocytosis pathway. Viral envelope fusion with the host cell membrane occurs in late endosomes, thus resulting in the release of vRNPs into the cytoplasm to begin viral genome transcription and replication. The final steps of the nairovirus replication cycle include particle assembly into the Golgi complex and their release from cells [1].

To date, HAZV has been isolated only in Western Pakistan from six *Ixodes redikorsevi* ticks collected from the Hazara District. Although anti-HAZV antibodies were found in a few serum samples from rodents and humans in Pakistan, no cases of human and animal infections have been reported, and the natural host of the virus is still unknown [1,2,3,4]. In the last decade, however, increasing interest has been shown in HAZV for its similarities—at structural, molecular and biological levels—with the Crimean-Congo hemorrhagic fever virus (CCHFV), the most relevant member of the *Nairoviridae* family [1,3,4]. Comparison of the NP and part of the L genomic segments of HAZV with different CCHFV strains reveals sequence identity of about 59% and 70%, respectively [3,5]. Moreover, the infection of mice with HAZV induces the production of cross-reacting antibodies to CCHFV, thus allowing the classification of both viruses in the same serogroup [4].

Unlike HAZV, CCHFV is highly pathogenic to humans and is classified in the risk group 4 of human pathogens [6]. In humans and animals, CCHFV infection is generally subclinical. However, roughly 20% of human infections are associated with a mild non-specific fever that can evolve to a severe hemorrhagic syndrome, with a fatality rate of 5–30% [7]. The death of patients is generally attributed to a disseminated intravascular coagulopathy (DIC), shock and/or multiorgan organ failure [8]. Humans can become infected, not only through the blood meal of tick vectors, but also after contact with contaminated blood, feces and tissues of viremic animals or humans [3,4,5,6,7].

However, vaccines and specific antiviral therapies for CCHF treatment are not yet available. Ribavirin, a purine nucleoside analogue targeting the RdRP of several RNA viruses, is the only antiviral recommended by the WHO (World Health Organization) and CDC (Centers for Disease Control and Prevention). However, the exact mechanism of its action against CCHF and the clinical efficacy remain to be clarified [7].

Considering that CCHFV is endemic in more than 30 countries in Asia, Africa, the Middle East, Southern and Southeastern Europe, and the Southern districts of Russia, it is considered as a possible cause of a global health emergency [7]. For this reason, in 2018 the WHO included CCHFV among the emerging infectious disease agents for which the development of effective prevention and therapeutic interventions is a priority [9].

Natural products from plant extracts, as well as from marine organisms and microorganisms, are currently considered a major source of potential antimicrobial agents, with a wide spectrum of action and showing minimal side effects and good tolerability [10]. In this context, extracts of the American cranberry (*Vaccinum macrocarpon* Aiton, *Ericaceae*), have been reported to possess antimicrobial activities by interfering with the attachment of pathogens to host tissue surfaces [11,12,13]. We recently observed that *V. macrocarpon* extracts show a virucidal activity against both influenza viruses A and B (IVA and IVB) and herpes simplex virus types 1 and 2 (HSV-1 and HSV-2) and showed that the antiviral efficacy of the plant extract was related to the content of A-type proanthocyanidins (PAC-A) [14,15]. It is also worth noting that antiviral activities of different cranberry extracts, such as juices and concentrates, have been reported against Reovirus, Rotavirus, Enterovirus, and polioviruses [13,14,15,16], thus suggesting the occurrence of cranberry constituents capable of broad-spectrum antiviral activity.

To contribute to the development of new therapeutic approaches for the treatment of CCHFV infection, here we use HAZV as a surrogate of CCHFV to investigate the in vitro antiviral activity of a cranberry extract characterized by a high content of PAC-A. The obtained results indicate that this cranberry extract exerts an anti-HAZV activity by targeting the adsorption phase of the virus replication cycle and as a consequence of a direct interaction with virus particles that abrogated their ability to bind cell receptors on target cells. Finally, we observed that the cranberry extract inhibits CCHFV infection in vitro. Together, our results suggest that this cranberry extract is a promising candidate to develop new antiviral strategies for the treatment of CCHFV infections.

## 2. Results

### 2.1. Cranberry Extract Targets Early Phases of the HAZV Replication Cycle

**Figure 1:**
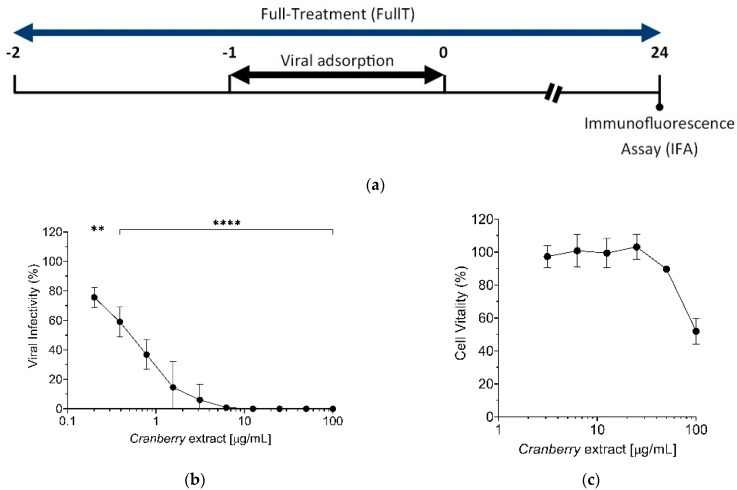
(**a**) Schematic representation of the “full-treatment” experiment; (**b**) Vero cells were treated with the cranberry extract at different concentrations throughout the experiment; the infection was conducted with HAZV at an MOI of 0.005 FFU/cell; the virus infectivity was evaluated by counting the IFA-positive HAZV-infected cells and then expressed as a % to the positive control of infection (untreated, set at the 100% value). (**c**) Cytotoxicity of the cranberry extract. Vero cells were treated with increasing concentrations of extract and cytotoxicity was evaluated after 24 h using the MTT assay. Data shown are the mean ± SD (standard deviation) of three independent experiments performed in quadruplicate. ** *p* < 0.01; **** *p* < 0.0001.

To evaluate the effect of the cranberry extract on HAZV replication, Vero cells were pre-treated with the extract for one hour before infection, then infected with HAZV. In addition to the pre-treatment, cells were continuously exposed to the cranberry extract (full treatment) during all the steps (Figure 1a). Thus, the rate of infection was evaluated by immunofluorescence (IFA) at 24 h post infection (p.i.). Cranberry extract inhibited HAZV infection in a dose-dependent manner (Figure 1b, Supplementary Figure S1) with an EC_50_ value of 0.6 μg/mL. Moreover, all the tested concentrations of the cranberry extract were not cytotoxic for Vero cells, since MTT assays measured a 50% cytotoxic concentration (CC_50_) > 100 μg/mL (Figure 1c), with Selective Index (SI) > 167, which indicated that the observed antiviral activity was specifically due to an inhibition of viral infection and not to cytotoxic effects.

**Figure 2:**
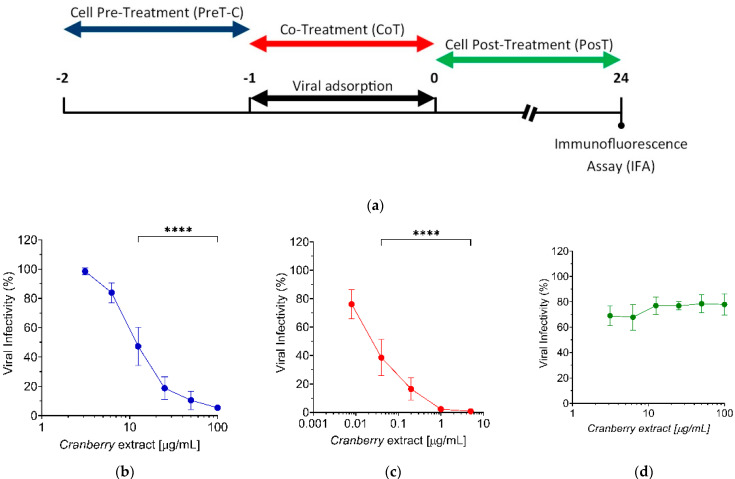
(**a**) Schematic representation of “time-of-addition” experiments. (**b**–**d**) The cranberry extract inhibits HAZV infection acting at early stage of the HAZV replication cycle. Vero cells were treated with extract at increasing concentrations (0, 3.125, 6.25, 12.5, 25, 50, and 100 μg/mL in (**b**,**d**) and 0.008, 0.04, 0.2, 1, and 5 μg/mL in (**c**)) (**b**) 1 h before infection (PreT-C), (**c**) during infection (CoT), or (**d**) immediately after infection (PosT) with HAZV at an MOI of 0.05 FFU/cell. At 24 h p.i., viral infectivity was determined by IFA. The virus infectivity was expressed as a % of the positive control of infection (untreated, set at the 100% value). Data represent mean ± SD of three independent experiments performed in triplicate. **** *p* < 0.0001.

To determine the stage of HAZV replication cycle at which the cranberry extract carried out the inhibitory activity, we performed three different time-of-addition treatments, as summarized in Figure 2a. Cells were treated with different concentrations of the cranberry extract 1 h before the infection (pre-adsorption stage, from −2 to −1, PreT-C) and/or during the infection (adsorption stage, from −1 to 0, CoT) and/or immediately after the infection (post-adsorption stage, from 0 to +24 h p.i., PosT). While the full treatment assessed the overall anti-HAZV activity of the cranberry extract, the PreT-C allowed for the evaluation of the ability of the cranberry extract to prevent HAZV infection by affecting the virus’s cell receptors. Moreover, the Co-T could provide insight into the capacity of the cranberry extract to interfere with the attachment and/or entry of HAZV into host cells. Finally, the PosT could indicate whether the cranberry extract can negatively affect biosynthetic events in the progression of the HAZV replication cycle after the binding/entry phases.

To this end, Vero cells were treated and infected according to the three treatments described, and at 24 h p.i., the number of infected cells was determined by immunofluorescence (IFA). As shown in Figure 2b,c, concentration-dependent inhibitory activity of the cranberry extract was observed in PreT-C and CoT treatments, while the HAZV infection rate was only weakly inhibited in the PosT schedule. Clearly, the cranberry extract inhibited virus infection by targeting early stages of the HAZV replication cycle. It is worth noting that, while in PreT-C and PosT experiments, cells were exposed to 3.125, 6.25, 12.5, 25, 50, and 100 μg/mL of the cranberry extract. In the case of CoT experiments, the concentrations that allowed for quantification of the inhibition of HAZV infection were lower, at 0.008, 0.04, 0.2, 1, and 5 μg/mL, thus indicating that the anti-HAZV potency of the cranberry extract was higher in CoT than that observed in the PreT-C schedule. The measurement of EC_50_ values confirmed that the cranberry extract was ~550-fold more potent when added during the adsorption/entry phase (EC_50_ CoT = 0.02 μg/mL), than before HAZV infection (EC_50_ PreT-C = 11.08 μg/mL), with a very high favourable SI > 5000. These results suggested that to achieve the strongest effect on HAZV infection rate, the cranberry extract should be added together with the infecting virus, thereby leading to an interference with the attachment and/or entry phases.

Taken together, these results indicate that the cranberry extract inhibited early stages of the HAZV replication cycle, such as during the initial interaction between viral envelope glycoproteins and cell receptors or by affecting the subsequent virus entry into the cytosol of target cells.

### 2.2. Cranberry Extract Inhibits HAZV Attachment to Target Cells

**Figure 3:**
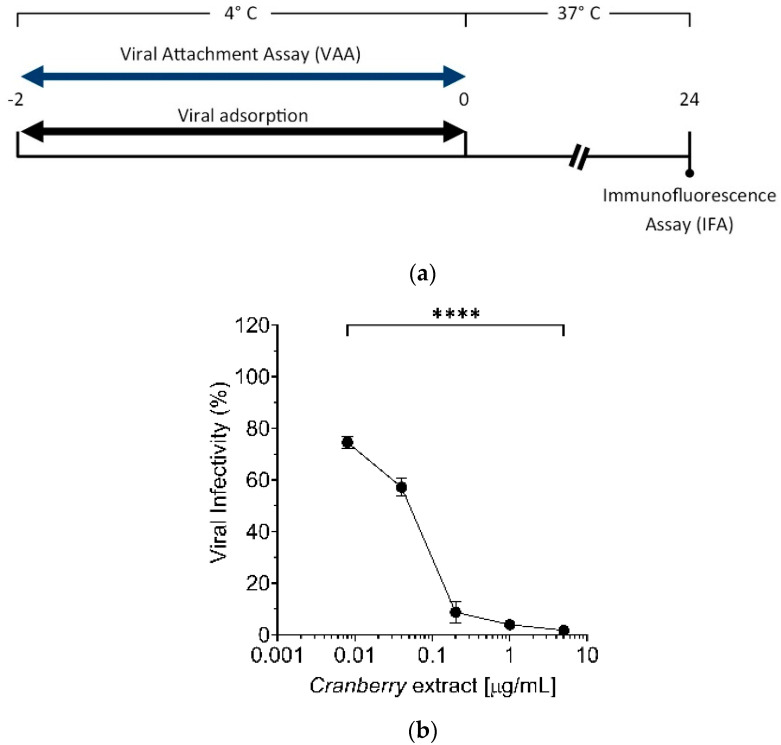
(**a**) Schematic representation of the Viral Attachment Assay (VAA); (**b**) HAZV attachment to target cells is prevented by cranberry extract. Precooled Vero cells were infected with HAZV at an MOI of 0.05 FFU/cell in the absence or in the presence of different concentrations of extract (0.008, 0.04, 0.2, 1, and 5 μg/mL) for 2 h at 4 °C (a condition known to allow the attachment to cells surface and prevent viral internalization). Then cell monolayers were washed with PBS and incubated with complete growth medium at 37 °C. At 24 h p.i., infected cells were detected by IFA. Viral infectivity was expressed as a % to the positive control of infection (untreated, set at the 100% value). The data shown represent mean ± SD of three independent experiments performed in triplicate. **** *p* < 0.0001.

Using the results obtained from the “time-of-addition” experiments, we investigated in depth the cranberry extract’s antiviral activity at the early stages of HAZV infection. To this end, a selective Viral Attachment Assay (VAA) was adopted to determine whether the cranberry extract could interfere with the initial adsorption of viral particles to the surface of target cells (Figure 3a). Thus, precooled Vero cells were therefore infected with HAZV in the presence of different concentrations of cranberry extract for 2 h at 4 °C to allow the attachment of viral particles to the cell surface, while preventing their entry into target cells. As a positive control of infection, cells were infected without the cranberry extract. After the attachment phase (2 h at 4 °C), the cranberry extract and unbound virions were removed by consecutive washings, and cells were shifted at 37 °C in standard grow conditions. At 24 h p.i., the number of infected cells was measured by IFA to correlate the percentage of infected cells with the rate of virus binding. As shown in Figure 3b, we observed a clear dose-response reduction of the number of infected cells, comparable to the CoT, thus indicating that cranberry extract indeed inhibited HAZV attachment.

### 2.3. Virucidal Activity of the Cranberry Extract against HAZV Particles

**Figure 4:**
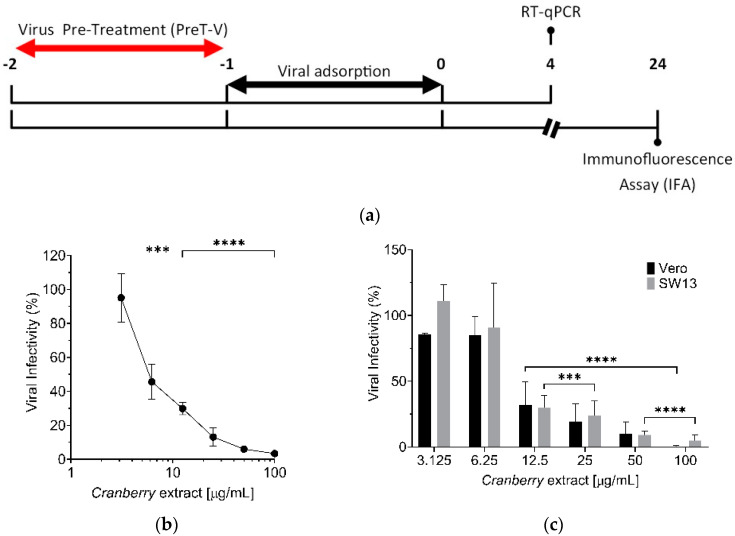
(**a**) Schematic representation of the PreT-V assay; (**b**) pre-incubation of HAZV virus particles with cranberry extract reduces viral infectivity. HAZV (10^3^ FFU) was incubated for 1 h at 37 °C without or with different concentrations of the extract (3.125, 6.25, 12.5, 25, 50, and 100 μg/mL). Then, the residual infectivity of mixtures was measured on Vero cell monolayers. At 24 h p.i., viral infectivity was determined, evaluating the number of infected cells by immunofluorescence. The reduction of viral infectivity was expressed as a percentage of the positive control of infection (untreated, set at 100%). (**c**) Evaluation of HAZV internalization in Vero and SW13 cells infected at an MOI of 1 FFU/cell. Viral particles were subjected to PreT-V before the infection of precooled Vero and SW13 cells. After 4 h p.i., cells were collected, and the number of internalized HAZV virions was quantified by RT-qPCR and normalized on the control (untreated cells). The data show means ± SD of three independent experiments performed in triplicate. *** *p* < 0.001; **** *p* < 0.0001.

Based on the above results, we then evaluated whether the cranberry extract exhibited a direct virucidal activity against HAZV particles. To verify this hypothesis, a Pre-Treatment (PreT-V) of HAZV particles with different concentrations of the extract (3.125, 6.25, 12.5, 25, 50, and 100 μg/mL) was performed for 1 h at 37 °C, as summarized in Figure 4a. Then, the residual HAZV infectivity was measured by infecting Vero cell monolayers with serial dilutions of the virus/extract mixtures. As shown in Figure 4b, the PreT-V of HAZV particles with the cranberry extract determined a concentration-dependent reduction of viral infectivity with an EC_50_ of 6.0 μg/mL, thus indicating the ability of the cranberry extract to interact with HAZV virions in such a way as to impair their subsequent attachment to target cells.

To further investigate how the cranberry extract interferes with HAZV infection, a different assessment of its ability to affect virus infectivity was performed by measuring the rate of internalized HAZV genomes in cells using real-time Reverse Transcriptase Quantitative Polymerase Chain Reaction (RT-qPCR). This assay was undertaken to exclude any confounding effect on the HAZV infection rate that might be due to the subsequent steps of the replication cycle.

For this purpose, HAZV particles were subjected to a PreT-V treatment, then precooled Vero cells were infected with HAZV at + 4 °C for 1 h (allowing virus binding but blocking the internalization), as reported in the material and methods section. At 4 h p.i., in which cells were incubated at 37 °C to start the internalization of bounded virions, infected cells were harvested, and total RNA was purified and submitted to RT-qPCR. Under these experimental conditions, the HAZV RNA replication phase was not yet started, and thus the measured viral RNA content corresponded to genomes of internalized virions (Figure 4a). RT-qPCR results of this modified PreT-V assay showed that the cranberry extract reduced the number of internalized HAZV genome copies in a concentration-dependent manner, thus confirming its ability to meddle in the binding of virions to cell receptors on the surface of target cells (Figure 4c). Moreover, this effect was also observed in a different cell model, the human adeno carcinoma SW13 cell line, thus establishing that the inhibitory activity of the cranberry extract on HAZV attachment is cell-type independent (Figure 4c).

Taken together, these results suggest that the overall antiviral activity of the cranberry extract against HAZV stems mainly from its ability to affect virion particles, thus impairing their attachment to host cells and preventing virus infection.

### 2.4. Cranberry Extract Inhibits CCHFV Infection

**Figure 5:**
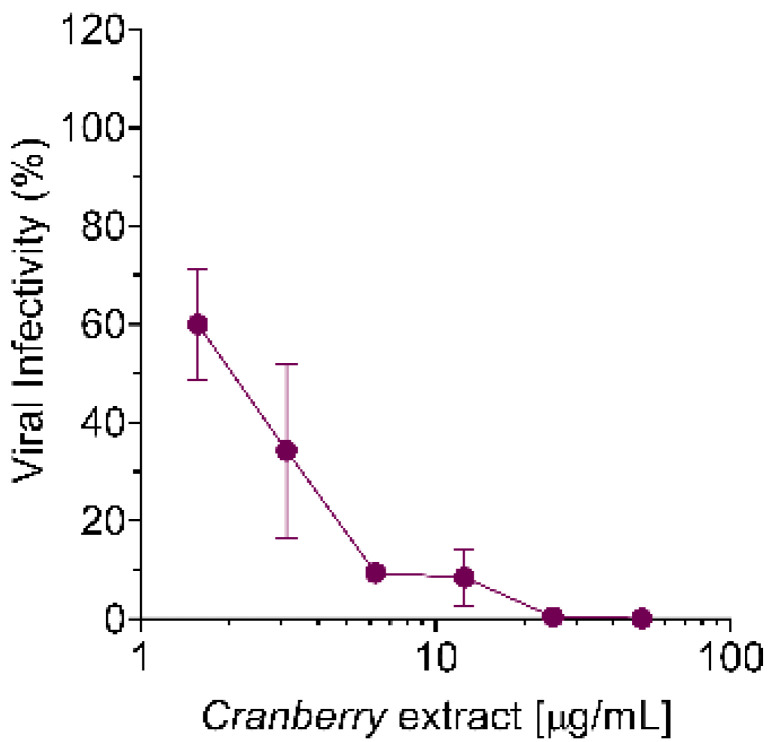
Cranberry extract inhibits CCHFV infection. Vero cells were treated for 1 h with extract at increasing concentrations (0, 3.125, 6.25, 12.5, 25, 50, and 100 μg/mL) during infection (CoT) with CCHFV at an MOI of 0.05 FFU/cell. Then, the virus inoculum was removed, and at 24 h p.i., the viral infectivity rate was determined by IFA. The virus infectivity was expressed as a % of the positive control of infection (untreated, set at the 100% value). Data represent mean ± SD of three independent experiments performed in triplicate.

To assess the effect of the cranberry extract on CCHFV infection, Vero cells were infected with CCHFV in the presence of different concentrations of cranberry extract for 1 h (CoT). Thereafter, the medium was exchanged, and 24 h later, cells were fixed and stained for CCHFV NP protein. As shown in Figure 5, the cranberry extract produced a clear concentration-dependent reduction of the CCHFV infectivity rate, with an EC_50_ of 2.01 ug/mL.

Taken together, the results of the different antiviral assays indicate that the cranberry extract can inhibit the infection of different nairoviruses, such as HAZV and CCHFV.

## 3. Discussion

Over the past 50 years, the spread of several arthropod-borne viruses has led to countless outbreaks and epidemics around the world. The recent increase in the geographical spread of arbovirosis has been driven by rapid urbanization, deforestation, climate change, and an increase in international travel and trade. Together, climate and environmental changes of natural or anthropic origin have led to the expansion of these viruses’ vectors into new territories, thus making contacts between humans and vectors themselves more frequent [7,10].

In this context, despite the recent re-emergence/emergence of CCHF in new territories, and its consequent inclusion in the research priority list by the WHO in 2018, no vaccine has been developed or approved, and there are still no specific and effective antiviral agents available for the treatment of the disease as, so far, very few studies have addressed the discovery of antivirals against CCHFV [9]. Therefore, to reduce the CCHF burden worldwide, the discovery and development of effective anti-CCHFV agents is urgent and mandatory for public health. For this purpose, the selection of bioactive natural products seems to be an encouraging approach. Among a large number of natural products evaluated for antiviral properties, the extract of American cranberry is an interesting candidate [10,17]. This extract contains high levels of A-type proanthocyanidins (PACs-A), a class of polyphenols endowed with broad-spectrum antiviral activities against viruses highly different in their structure and replication strategies [10,17,18,19]. In this regard, enriched polyphenol extracts of natural origin and natural polyphenols have been described to possess antiviral activity against viruses responsible for hemorrhagic fevers [10,20]. For example, the epigallocatechin gallate (EGCG) and the polyphenol-rich extracts from *Psiloxylon mauritianum* and *Aphloia theiformis*, two indigenous medicinal plants from an island in the Mascarene Archipelago (Indian Ocean), have been observed to carry out a potent antiviral activity against the Dengue virus (DENV), an arbovirus of the *Flaviviridae* family causing a hemorrhagic fever, which is widespread in tropical and subtropical countries [10,21,22]. Moreover, the Lassa virus (LASV), an arenavirus responsible for a severe hemorrhagic fever, which is transmitted to humans by rodents, has been shown to be sensitive to the inhibitory activity of tangeretin, a polyphenol (polymethoxylated flavone) characteristic of the peel of fruits of the genus *Citrus*, such as mandarins and oranges. Tangeretin, specifically, inhibits the internalization of LASV particles into host cells by blocking the fusion event mediated by viral envelope glycoproteins with late endosome membranes [23]. In contrast, ECGC and the polyphenol-rich extracts of *P. mauritianum* and *A. theiformis* prevent Dengue virus replication by interacting directly with the surface of the virions and therefore inhibiting their initial attachment to host cells [10,21,22,23].

Based on these facts, we carried out an in vitro study to evaluate the anti-CCHFV activity of cranberry extract using HAZV, a faithful and safe surrogate of CCHFV, to overcome the limitations of requiring a biosafety level 4 facility [3,4,5,6,24].

Time-of-addition experiments demonstrated that the cranberry extract acts on the early steps of the HAZV replication cycle, in accordance with our previous observations regarding IVA, IVB, HSV-1, and HSV-2 [14,15]. Early stages of HAZV infection include attachment to the cell surface and entry via endocytosis. To discriminate which of these steps is affected by cranberry extract, selective Viral Attachment Assay (VAA) and virus internalization experiments were conducted. Results suggest that the cranberry extract inhibits HAZV attachment to the cell surface, as reported for IV and HSV [14,15]. However, in the case of influenza viruses, it has been observed that this cranberry extracts affect not only the phase of virus attachment to the cell surface, but also the subsequent internalization [14]. This suggests that the antiviral activity of the cranberry extract may be different depending on the type of glycoproteins of the viral envelope or perhaps on the specific mechanism of the virus entry into target cells.

Nevertheless, the mechanism of action of the cranberry extract seems to be the same for all the viruses examined so far, as it stems from the ability of the cranberry extract to interact directly with viral particles, thus exerting a virucidal activity. In fact, even for HAZV, we observed a dose-dependent virucidal activity of the cranberry extract, with an EC_50_ of 6.0 µg/mL (Figure 4b). Moreover, the effect was independent of the host cell type used for infection. Interactions of the cranberry extract with the surface of virus particles, in particular with those envelope glycoproteins responsible for attachment and entry, may affect their functions in the initial phases of the viral replication cycles, thus explaining the majority of the overall anti-HAZV activity of the cranberry extract.

However, we also observed a reduction of HAZV infectivity in the PreT-C schedule of treatment, which required the incubation of the cranberry extract with target cells prior to virus infection (Figure 2b). This inhibitory effect could be explained by a possible binding of components of the cranberry extract to cell surface proteins, including viral receptors. Through such interactions, the cranberry extract’s components likely alter and/or mask cell receptor binding sites for HAZV glycoproteins, thus reducing the virus attachment to target cells. A similar mechanism of action of cranberry extract has been reported for the bovine reovirus type 3, a model of enteric viruses [16]. On the other hand, in the “Co-Treatment” (CoT), the simultaneous action of the cranberry extract on the envelope glycoproteins of viral particles and cell receptors maximized its antiviral effect, with an EC_50_ ~ 550 times lower than that of the PreT-C (Figure 2c), therefore supporting the view that the major contribution to the overall antiviral activity of the extract involves an alteration of the surface of viral particles.

In this regard, we previously observed that the cranberry extract interacts in vitro with the ectodomains of both the Hemagglutin (HA) of IVA and the gD of HSV-1 and HSV-2, as indicated by the alteration of the electrophoretic mobility of the corresponding recombinant viral proteins upon incubation with the extract [14,15]. A specific component of the cranberry extract, the PACs-A dimers (PACs-A2), could be responsible for such interactions, since an incubation of IVA or IVB particles with purified PACs-A2 caused a complete loss of their infectivity, thus indicating their ability to bind IV particles, thereby impairing their efficient adsorption to target cells [14]. However, it is not yet clear whether this virucidal activity results from the PACs-A2′s “coating” of the entire viral envelope glycoprotein array or whether it is due to the binding of PACs-A2 to specific viral glycoproteins; as observed for the whole cranberry extract, the binding of PACs-A2 to viral glycoproteins may compromise their essential functions in the early stages of infection. The known ability of polyphenols to bind proteins and generate non-functional aggregates sustains a role of PACs-A2 in altering viral glycoprotein functions [14,15,25]. Furthermore, the PACs content of this cranberry extract has also been shown to be responsible for the anti-adhesive activity of the extract against bacteria, such as uropathogenic strains of *E. coli* [12]. Considering that the cranberry extract used in this study was characterized by a high percentage of PACs-A2, we can hypothesize that the inhibition of the HAZV attachment to cell surfaces observed in this study may depend on PACs-A2′s interaction with glycoproteins Gn and Gc on the surface of HAZV particles. To verify this hypothesis, the in vitro anti-HAZV activity of purified PACs-A2 will be tested in the future to confirm that, even for *Nairoviridae*, PACs-A dimers are the active components responsible of the majority of the overall antiviral activity of the extract.

Finally, we confirmed the antiviral activity of the extract against CCHFV (Figure 5), though with an EC_50_ higher than that measured against HAZV. This difference in the EC_50_ values between the two nairoviruses might be due to a differential efficiency in virus attachment to target cells. In this regard, though the attachment/receptor factors of CCHFV and HAZV are still unknown, there is evidence suggesting the involvement of different cellular molecules [26].

In conclusion, although further investigations are required to confirm the bioactive components of the extract responsible for the observed anti-nairovirus activity, the results of this study suggest the cranberry extract as a promising candidate for the development of antivirals against CCHFV. More importantly, the broad-spectrum antiviral activity of cranberry extract and of other plant extracts characterized by a high content of PACs-A2 could be valuable to design and develop new antivirals against many current viral diseases and future viral threats.

## 4. Materials and Methods

### 4.1. V. macrocarpon Extract

The cranberry (*V. macrocarpon* Aiton) extract was obtained from cranberry fruits using an optimized procedure (patent pending). The authentication of the extract was performed by Alkemist Labs (Garden Grove, CA, USA) (Supplementary Figure S2). The extract was resuspended in Dulbecco’s modified Eagle Medium (DMEM; Life Technologies, Monza, Italy) at the final concentration of 1 mg/mL. Aliquots of stock solution were stored in 1.5 mL vials at −80 °C until use.

### 4.2. Quantification of Total and A-Type Proanthocyanidins

To determine the total proanthocyanidin (PAC) content, the Brunswick Labs p-dimethylaminocinnamaldehyde (BL-DMAC) assay was performed according to the method described by Prior et al. [27]. Total PACs were quantified using an external calibration curve, generated using a pure PAC-A2 standard (Sigma-Aldrich, Milan, Italy). The quantification was performed in triplicate within the linear range of the calibration curve (5–30 µg/mL).

The PAC-A content of the cranberry extract was determined by HPLC-ESI-MS/MS, as previously described [12]. The identification of PACs (from dimers to pentamers) was performed via multiple reaction monitoring (MRM) mass spectrometry of the following molecular ions [M-H]^-^: 575 *m*/*z* for A-type dimers and 577 *m*/*z* for B-type dimers; 861 *m*/*z* for 2A-type, 863 *m*/*z* for 1A-type and 2B-type trimers, and 865 *m*/*z* for 2B-type trimers; 1147 *m*/*z* for 3A-type, 1149 *m*/*z* for 2A-type and 1B-type, 1151 *m*/*z* for 1A-type and 2B-type, and 1153 *m*/*z* for 3B-type tetramers; 1433 *m*/*z* for 4A-type, 1435 *m*/*z* for 3A-type and 1B-type, 1437 *m*/*z* for 2A-type and 2B-type, 1439 *m*/*z* for 1A-type and 3B-type, and 1441 *m*/*z* for 4B-type pentamers (supplementary results).

### 4.3. Cells, Culture Conditions and Viruses

African green monkey kidney cells (Vero, ATCC^®^ CCL-81™) were cultured in DMEM supplemented with 10% fetal bovine serum (FBS; Life Technologies, Monza, Italy) inactivated for 30 min at 56 °C (FBSi). Vero cells were maintained in a humidified atmosphere at 37 °C with 5% CO_2_.

Human adrenal cortex adeno carcinoma cells (SW13, ATCC^®^ CCL-105™) were cultured in Leibovitz’s L-15 Medium (Life Technologies, Monza, Italy) supplemented with 10% FBSi. SW13 cells were maintained in a sealed flask with a standard atmosphere at 37 °C.

The HAZV strain JC280 and CCHFV strain IbAr10200 used in these experiments were amplified on SW13 cells. To this end, 6 × 10^6^ SW13 cells were seeded in a T75 flask (Sarstedt, Verona, Italy). After 24 h, cells were infected with HAZV or CCHFV at the multiplicity of infection (MOI) of 0.1 fluorescent focus-forming units (FFU)/cell in Leibovitz’s L-15 medium. After 1 h incubation at 37 °C, the viral inoculum was removed and cells were washed twice with phosphate-buffered saline 1X (PBS 1X; Life Technologies, Monza, Italy) and incubated with culture medium supplemented with 2% FBSi. Cells were then placed at 37 °C, and 48 h later, the supernatant was collected and centrifuged at 3000 rpm for 5 min at 4 °C to remove any cellular debris. Aliquots of the viral stock were stored at −80 °C until use. Experiments with CCHFV were performed in the BSL-4 laboratory at the Public Health Agency of Sweden.

### 4.4. Viral Titration

The virus was titrated by immunofluorescence assay (IFA) in Vero cells as previously described [26]. Briefly, Vero cells were infected (1 h at 37 °C) with serial 10-fold dilutions of the viral stock performed in DMEM without FBSi. Thereafter, cells were washed in PBS 1X and DMEM medium with 2% of FBSi added. At 24 h post-infection (h p.i.), the viral titer was determined by immunofluorescence assay (IFA), as described below. Viral stocks usually reached 10^6^ FFU/mL.

For the immunofluorescence assay (IFA), the supernatant was removed from infected cell cultures, and cell monolayers were then washed with PBS 1X and fixed using precooled acetone:methanol (1:1) solution for 45 min at −20 °C. After this, once the fixing solution was removed, cells were washed with PBS 1X, and nonspecific sites were blocked with PBS 1X/2.5% BSA (*Bovine Serum Albumin*; Merck, Darmstadt, Germany) for 1 h at room temperature (RT). Subsequently, cells were incubated for 1 h and 30 min at 37 °C with an in-house-developed anti-nucleoprotein antibody (α-NP), diluted 1:200 in PBS 1X/2.5%BSA/0.1%Tween 20 (Merck). After this incubation, cells were washed with PBS 1X and incubated for 1 h at 37 °C in the dark with Alexa Fluor^TM^ 488 goat anti-rabbit IgG (Thermo Fisher Scientific, Monza, Italy), diluted 1:800 in PBS 1X/2.5% BSA/0.1% Tween 20/DAPI (Merck) 1:1000. The DAPI staining was used to counterstain the cells’ nuclei.

Foci formed by the viral infection were counted using a fluorescence microscope (Zeiss^®^, Milano, Italy), and viral titer was expressed as FFU/mL.

### 4.5. Antiviral Assays

The antiviral activity of cranberry extract against the viral replication cycle was determined using “time-of-addition” assays, as summarized in the scheme depicted in Figure 2 [14]. Briefly, Vero cells were seeded in a 96-well plate (2 × 10^4^ cells/well). The following day, different extract concentrations (0, 3.125, 6.25, 12.5, 25, 50, and 100 μg/mL) were added 1 h before infection (cells “Pre-Treatment”, PreT-C) or 1 h after infection (cells “Post-Treatment”, PosT). In the “Co-Treatment” of cells and HAZV with cranberry extract experiment (CoT), cells were exposed during virus adsorption to different concentrations of extract (0, 0.008, 0.04, 0.2, 1, and 5 μg/mL). In the full treatment, Vero cells were treated with extract 1 h before the infection, during the HAZV adhesion, and after the infection until the end of the experiment. In all the assays, the cells were infected with serial 10-fold dilutions, prepared starting from non-diluted virus (10^5^ FFU/well) for 1 h at 37 °C and then washed with PBS 1X to remove unbound virions. At 24 h p.i., viral load was determined by IFA as described above. In the CoT experiment to test the antiviral activity of the cranberry extract against CCHFV, we used the following concentrations of the extract: 0, 3.125, 6.25, 12.5, 25, 50, and 100 μg/mL and an MOI of 0.05 FFU/cell. For the IFA, we employed an in-house-developed anti-nucleoprotein antibody (α-NP), following the protocol above described.

For the “Viral Attachment Assay” (VAA), Vero cells were seeded as for PreT-V and “time-of-addition” assays [14]. The following day, cultures were cooled for 20 min at 4 °C and then washed three times with cold PBS 1X. After that, precooled Vero cells were infected at 4 °C for 2 h with 10-fold serial dilutions of the viral stock (10^5^ FFU/mL) in the presence of different concentrations of extract (0, 0.008, 0.04, 0.2, 1, and 5 μg/mL). After viral adsorption, cells were washed twice with PBS 1X at RT and incubated with DMEM medium supplemented with 10% FBSi for 24 h at 37 °C. The following day, the medium was removed, and IFA was performed as described above.

The virucidal activity of cranberry extract against HAZV was determined using a “Viral Pre-Treatment” assay (PreT-V), as summarized in the scheme depicted in Figure 4a. To this end, Vero cells were seeded in a 96-well plate (2 × 10^4^ cells/well). The following day, different concentrations of extract (0, 3.125, 6.25, 12.5, 25, 50, and 100 μg/mL) were incubated with different HAZV aliquots (5 serial 10-fold dilutions, prepared starting from a stock virus, 10^5^ FFU/well) in DMEM without FBSi for 1 h at 37 °C. After this incubation period, extract/HAZV mixtures were diluted and added to cells for 1 h at 37 °C. After virus adsorption, cells were washed with PBS 1X, and DMEM medium with 10% FBSi was added. At 24 h p.i., viral infectivity was determined by IFA as described above.

### 4.6. Real-Time Quantitative Reverse Transcriptase Polymerase Chain Reaction (RT-qPCR)

Samples submitted to RT-qPCR were obtained from Vero and SW13 cells infected with HAZV, pre-treated with cranberry extract (Figure 4a). Briefly, Vero cells were seeded in a 12-well plate (2 × 10^5^ cells/well). The following day, precooled cells were infected with HAZV, previously treated with different concentrations of cranberry extract (3.125, 6.25, 12.5, 25, 50, and 100 μg/mL), at the MOI of 1 FFU/cell. At 1 h p.i., unbound virions were removed by washing cells with PBS 1X, and cells were incubated with DMEM medium supplemented with 10% FBSi at 37 °C. At 4 h p.i., cells were washed with PBS 1X and incubated with trypsin (Life Technologies, Monza, Italy). Once the cells detached from the wells, trypsin was neutralized with the same volume of DMEM supplemented with 10% FBSi. Cells were harvested by centrifugation at 6000 rpm for 7 min at RT and washed twice with PBS 1X. Finally, cells were centrifugated at 6000 rpm for 7 min at RT, supernatants were removed, and cell pellets were stored at −80 °C until total RNA extraction.

The total RNA, including genomic viral RNA, was extracted from cell pellets using the RNeasy Mini kit (Thermo Fisher Scientific, Monza, Italy) following the handbook’s instructions.

For the qRT-PCR, the RT reaction mix used comprises 1 μg of template RNA, 11 μL of 25 mM MgCl_2_, 5 μL of 10X Buffer without Mg^2+^, 10 μL of 2.5 mM dNTPs, 1.5 μL of RNAseIN, 1.5 μL of MuLV reverse transcriptase, and 2.5 μL of random hexamers. All reagents were purchased from Thermo Fisher Scientific (Monza, Italy). Water was used to fill up to a final total reaction volume for each sample of 50 μL. The cycling conditions were 25 °C for 10 min, 42 °C for 60 min, 95 °C for 3 min, and 4 °C.

Then, the real-time RT-qPCR was performed with an ABI PRISM^®^ 7000 Sequence Detection System (Applied Biosystems, Thermo Fisher Scientific, Monza, Italy) using primers and a probe targeting a portion of the S genomic segment of HAZV [26]. The real-time RT-qPCR reaction mix comprises 10 μL reaction TaqMan Universal PCR MasterMix (Thermo Fisher Scientific, Monza, Italy), 1 μL of each HAZV primers 18 μM, and 0.2 μL of HAZV probe 25 μM. Water was used to fill up to a total reaction volume of 15 μL, and finally 5 μL of the template cDNA was added. The cycling conditions used were those indicated by the manufactures’ instructions for TaqMan Universal PCR MasterMix.

For each single-well amplification reaction, a threshold cycle (Ct) was calculated in the exponential phase of amplification. A PCR product for the detection of HAZV, previously cloned in the pJET1.2/blunt plasmid using the CloneJET PCR cloning kit (Thermo Fisher Scientific, Monza, Italy), was used as a template to generate a standard curve to quantify the S fragment genomic copies in each cDNA sample.

### 4.7. Statistical Analysis

For each test, at least three independent biological replicates were performed. Statistical analysis was carried out with GraphPad Prism 8 software package (GraphPad Software, San Diego, CA, USA), employing the one-way ANOVA test. The threshold for statistical significance was *p* < 0.05. Details on sample size and *p* values are provided in the relevant figure or its legend. Curve fitting was performed to determine EC_50_ values using a sigmoidal 4PL model in GraphPad Prism 8 software.

## Data Availability

Publicly available datasets were analyzed in this study. This data can be found here: http://researchdata.cab.unipd.it/500/.

## Supplementary Materials

The following are available online at https://www.mdpi.com/article/10.3390/pathogens10081025/s1, Figure S1: Cranberry extract inhibits HAZV infection. Vero cells were treated with the cranberry extract at different concentrations throughout all the experiment, the infection was conducted with HAZV at a MOI of 0.005 FFU/cell; Figure S2: Authentication of the cranberry extract; Supplementary results: The cranberry extract contains a high percentage of A-Type Proanthocyanidins.
