# Supplementary material for: Cranberry (Vaccinium macrocarpon) Extract Impairs Nairovirus Infection by Inhibiting the Attachment to Target Cells
Source: Pathogens. 2021 Aug 13;10(8):1025. doi: 10.3390/pathogens10081025 (PMC8401317; doi:10.3390/pathogens10081025)
Supplement: Supplementary file 1 [file pathogens-10-01025-s001.zip › pathogens-1273808-supplementary.pdf]

pathogens

MDPI

Supplementary Materials

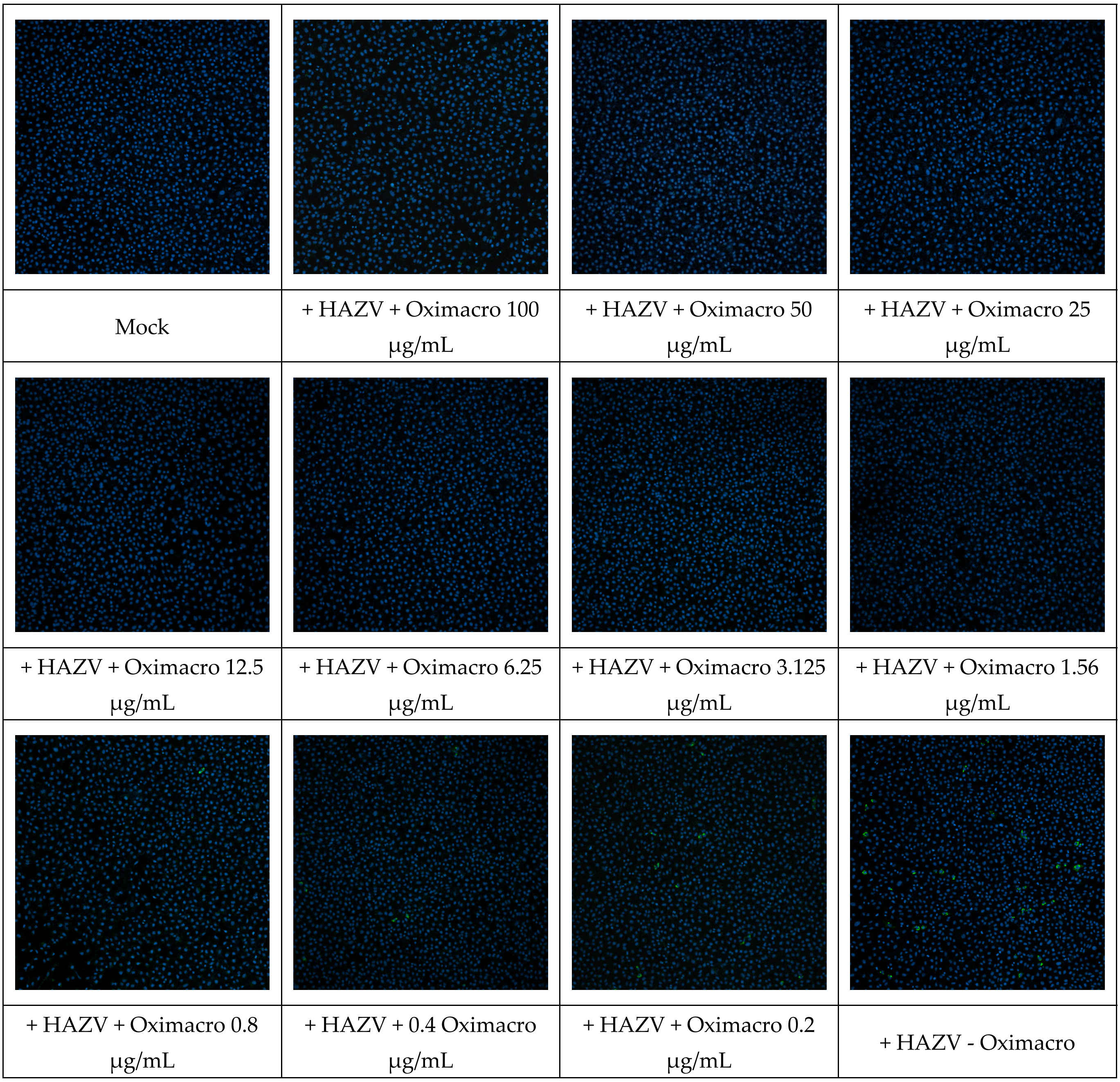

Figure S1. Cranberry extract inhibits HAZV infection. Vero cells were treated with the cranberry extract at different concentrations throughout all the experiment, the infection was conducted with HAZV at a MOI of 0.005 FFU/cell.

Work performed at:
Alkemist Labs
12661 Hoover Street
Garden Grove, CA 92841
714-754-HERB (4372)
714-668-9972 (FAX)
Sales@Alkemist.com
www.Alkemist.com

High Performance Thin-Layer Chromatography with Photo-Documentation

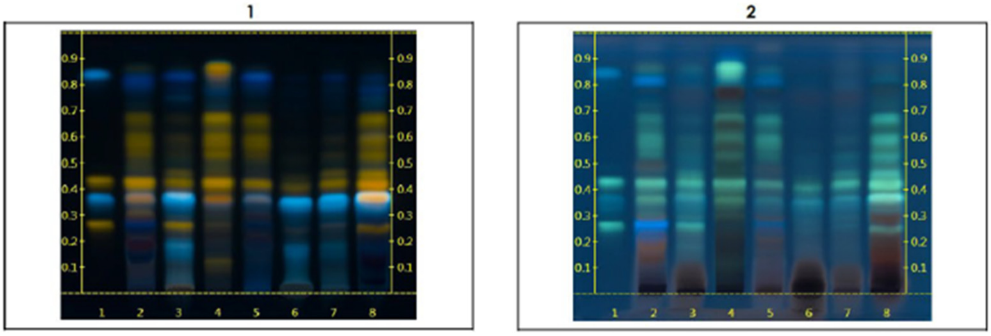

Plant Part:	Fruit Juice
Sample Received:	02/14/20
Sample Packaging:	Foil Pouch
Form of Botanical:	powdered extract
Appearance:	Fine powder
Lot Number:	(CRP01-191002) →Lane 4(4µl)
Sample:	20045XPH_1
Latin Name:	*Vaccinium macrocarpon* Aiton [Ericaceae]
Reference Sample:	Lane 2(10µl) (LQB15918AP1), Lane 8(10µl) (LQA17018NBTC1) Vaccinium sp. (fruit); Lane 3(10µl) (L11409SYN) Vaccinium macrocarpon (fruit); Lane 6(10µl) (KY06210AP1) Vaccinium corymbosum (fruit); Lane 7(10µl) (AAG18709UPL) Vaccinium angustifolium (fruit); held at Alkemist Labs, Garden Grove, CA.
Analyst:	A. Davis, N. Afendikova, M. Edwards, S. Kabbaj, N. Hoang, K. Tran, J. Lopez, J. Mares 131733
Sample Preparation:	1g+10mL water, sonicate/heat at 50°C for 30 min. SPE condition with 5mL Methanol, equilibrate with 5mL water, load, dry, elute with 1mL Methanol(2x)
Stationary Phase:	Silica gel 60, HPTLC plates
Mobile Phase:	ethyl acetate: Acetic acid: Formic Acid: Water [10/0.9/0.9/2]
Detection:	(1) Natural Product + Polyethylene Glycol, 366nm (Reich, E., 2007)<br>(2) 10% Sulfuric, 100°C, 2min, 366nm (Reich, E., 2007)
Reference Standard:	Lane 1(2µl) Chlorogenic Acid (11 1026/0, XSYN), Caffeic acid (1117/0, XSYN), Hyperoside (28 0702/0, XSYN), Rutin (A0348926, ACR), Methanol (0000206697, BDH)
Reference Source:	Method Developed by Alkemist Labs<br>IDT-SOP-72-01

Comments & Conclusions: Lane 4 is the test sample OXIMACRO/CRANBERRY EXT (CRP01-191002) Lanes 2, 3, 6, 7, 8 are the reference samples used for comparison. This test sample, OXIMACRO/CRANBERRY EXT (CRP01-191002) is consistent with the chromatographic profile of the reference samples of Vaccinium sp., used above. **This test sample OXIMACRO/CRANBERRY EXT (CRP01-191002) has characteristics of a customized extract derived from *Vaccinium* sp., juice.**

Figure S2. Authentication of the cranberry extract.

Supplementary results: The cranberry extract contains a high percentage of A-Type Proanthocyanidins.

The DMAC assay on the cranberry extract showed a 376.98 mg g^{-1} (± 0.63) total PAC content which was characterized by 57% dimeric PAC-A; 21% trimeric PAC 1A,1B; 1% pentameric PAC 3A,1B, 11% pentameric PAC 2A,2B and 1% pentameric 1A,3B. These results indicate a high content of PAC-A of the cranberry extract used for this study.
